# Retinal Diseases: Mechanisms, Diagnosis and Treatments—From Mechanistic Insights to Personalized Retina Care

**DOI:** 10.3390/jpm16080404

**Published:** 2026-07-28

**Authors:** Horia Tudor Stanca

**Affiliations:** Ophthalmology Department, “Carol Davila” University of Medicine and Pharmacy, 020021 Bucharest, Romania; horia.stanca@umfcd.ro

The past decade has witnessed remarkable progress in the understanding and management of retinal diseases. Rapid advances in retinal imaging [1], molecular diagnostics [2], genetics [3], pharmacotherapy [4], and vitreoretinal surgery [5] have transformed both clinical practice and research. Precision medicine has become an increasingly realistic goal, with clinicians now able to combine multimodal imaging [6,7], genomic information, and individualized therapeutic approaches to improve diagnosis, prognosis, and treatment selection [8]. Nevertheless, retinal diseases remain among the leading causes of irreversible visual impairment worldwide [9], underscoring the need for continued innovation and multidisciplinary collaboration [10].

When we conceived this Special Issue, Retinal Diseases: Mechanisms, Diagnosis and Treatments, our objective was to provide a comprehensive forum for emerging concepts spanning disease mechanisms, diagnostic technologies, and therapeutic innovations. We sought contributions that would not only advance scientific knowledge but also facilitate the translation of research discoveries into clinical practice. The resulting collection successfully reflects the diversity and rapid evolution of contemporary retinal medicine.

The Special Issue opens with the work of Matar et al., who investigated longitudinal ellipsoid zone dynamics in patients receiving hydroxychloroquine therapy (contribution 1). Using quantitative optical coherence tomography (OCT) analysis, the authors demonstrate that subtle alterations of the ellipsoid zone may precede clinically recognized retinal toxicity. Their findings highlight the growing importance of objective imaging biomarkers for detecting retinal damage at its earliest stages and suggest that quantitative OCT metrics may improve screening strategies for hydroxychloroquine retinopathy [11].

Jo et al. provide valuable insight into neonatal retinal hemorrhages through fundus photography-based analysis (contribution 2). Their study demonstrates characteristic patterns of hemorrhage distribution and supports elevated central venous pressure during vaginal delivery as an important pathogenic mechanism. Equally reassuring is the observation that these hemorrhages resolve spontaneously without significant short-term visual consequences, providing useful information for clinicians involved in neonatal ophthalmic care.

The relationship between systemic disease and retinal structure is explored by Carollo et al., who examined associations between short-term blood pressure variability and choroidal thickness measured by swept-source OCT (contribution 3). Their findings reinforce the concept that the choroid represents a sensitive biomarker of systemic vascular health and further strengthen the growing role of ophthalmic imaging in cardiovascular risk assessment [12].

The increasing globalization of retinal research requires normative databases that account for ethnic variability [13]. Addressing this important issue, Bujor et al. compared OCT angiography metrics between healthy Chinese and Caucasian individuals (contribution 4). Their study identified significant differences in retinal microvascular parameters and foveal avascular zone morphology, emphasizing the necessity of population-specific reference values when interpreting OCTA findings and developing artificial intelligence algorithms.

Inherited retinal diseases continue to represent one of the most rapidly evolving areas of ophthalmology [14,15]. In their comprehensive review, Kang et al. summarize the remarkable evolution of genetic testing, from conventional sequencing approaches to next-generation sequencing, transcriptomics, structural variant analysis, and artificial intelligence-assisted interpretation (contribution 5). Importantly, the review highlights how molecular diagnosis has become indispensable for patient selection in gene therapy and clinical trials while also identifying persistent challenges regarding access, variant interpretation, and global implementation.

The review by Dumitrescu et al. focuses on fundus autofluorescence in diabetic retinopathy, emphasizing its expanding role within multimodal retinal imaging (contribution 6). Beyond its diagnostic utility, fundus autofluorescence provides valuable information regarding retinal pigment epithelium integrity, oxidative stress, photoreceptor damage, and visual prognosis [7]. As imaging technologies continue to evolve, such biomarkers may increasingly guide individualized treatment strategies for diabetic retinal disease.

Finally, Vasilijevic et al. investigated retinal perfusion in children with type 1 diabetes using OCT angiography (contribution 7). Although retinal vascular parameters remained largely preserved in young patients without clinical diabetic retinopathy [8], the study suggests that insulin delivery methods may influence early retinal microvascular health. These findings illustrate the importance of identifying subclinical biomarkers capable of detecting retinal alterations before irreversible diabetic complications develop.

Taken together, these seven contributions demonstrate how modern retinal research extends far beyond traditional clinical observation. Quantitative imaging, advanced genetics, and interdisciplinary investigation are reshaping our understanding of retinal diseases while creating opportunities for increasingly personalized patient care.

Despite these significant advances, important knowledge gaps remain. Early detection continues to represent one of the greatest challenges in retina. Many retinal disorders progress silently, with irreversible structural damage occurring before functional impairment becomes clinically apparent. Although OCT, OCT angiography, fundus autofluorescence, adaptive optics, and ultra-widefield imaging have dramatically improved disease visualization, many imaging biomarkers require further longitudinal validation before they can be routinely incorporated into clinical decision-making.

Similarly, artificial intelligence has emerged as one of the most promising technologies in ophthalmology, yet several obstacles limit widespread implementation. Robust external validation, algorithm transparency, standardized datasets, and seamless integration into clinical workflows remain essential prerequisites before AI-assisted diagnostics can achieve their full clinical potential.

Inherited retinal diseases illustrate another area where remarkable scientific progress coexists with substantial unmet need. While genetic testing now identifies the molecular basis of disease in an increasing proportion of patients, many individuals still lack a definitive diagnosis. Furthermore, effective gene-specific therapies remain available for only a limited number of inherited disorders. Continued advances in gene editing, RNA therapeutics, optogenetics, stem-cell therapies, and regenerative medicine will be critical for expanding treatment opportunities.

Likewise, retinal vascular diseases continue to present considerable therapeutic challenges. Although anti-vascular endothelial growth factor therapy has revolutionized the treatment of neovascular age-related macular degeneration, diabetic macular edema, and retinal vein occlusions, treatment burden remains high, and incomplete responses are common. Future therapies will likely combine longer-lasting pharmacologic agents, sustained drug-delivery systems, neuroprotective strategies, complement inhibition, and anti-fibrotic approaches to improve both efficacy and durability.

Importantly, this Special Issue contributes directly toward addressing many of these challenges. The included studies expand our understanding of quantitative imaging biomarkers, explore the interface between systemic and ocular disease, refine genetic diagnostics, improve knowledge of retinal vascular physiology, and advance personalized approaches to retinal care. Together, they exemplify the multidisciplinary nature of contemporary ophthalmic research.

Looking toward the future, retinal medicine is poised to enter an era characterized by the integration of multimodal imaging, genomics, artificial intelligence, and precision therapeutics [16,17]. Predictive models incorporating structural imaging, functional assessment, molecular profiling, and systemic risk factors may enable truly individualized disease prediction and management. Home-based retinal monitoring, teleophthalmology [18], wearable imaging technologies, and digital health platforms are also expected to transform patient follow-up, improving accessibility while reducing healthcare burden.

Future research should prioritize several key areas: the identification and validation of robust imaging and molecular biomarkers; the integration of multi-omics approaches with clinical phenotyping; the development of explainable and generalizable artificial intelligence systems; expansion of gene-editing and regenerative therapies; optimization of sustained ocular drug-delivery platforms; and the establishment of large international collaborative datasets with standardized imaging protocols and clinical outcome measures. Such initiatives will accelerate the translation of scientific discoveries into routine patient care while ensuring that advances benefit diverse populations worldwide.

In conclusion, this Special Issue illustrates the remarkable progress being achieved across the spectrum of retinal research. The contributions presented here deepen our understanding of disease mechanisms, demonstrate the power of advanced diagnostic technologies, and highlight emerging therapeutic opportunities that are rapidly reshaping clinical practice. We sincerely thank all authors for their outstanding contributions, the reviewers for their rigorous and constructive evaluations, and the editorial team of the Journal of Personalized Medicine for their invaluable support throughout the publication process. We hope this collection will serve as a valuable resource for clinicians and researchers alike, stimulate new collaborations, and inspire future investigations that continue to advance personalized retinal care and ultimately improve visual outcomes for patients around the world.
